# Isolation of *vanA*-Mediated Vancomycin-Resistant *Enterococcus faecalis* (ST1912/CC116) and *Enterococcus faecium* (ST80/CC17), *optrA*-Positive Linezolid-Resistant *E. faecalis* (ST32, ST1902) from Human Clinical Specimens in Bangladesh

**DOI:** 10.3390/antibiotics14030261

**Published:** 2025-03-04

**Authors:** Sangjukta Roy, Meiji Soe Aung, Shyamal Kumar Paul, Md. Nazmul Alam Khan, Syeda Anjuman Nasreen, Muhammad Saiful Hasan, Nazia Haque, Tridip Kanti Barman, Jobyda Khanam, Fardousi Akter Sathi, Shashwata Paul, Mohammad Ibrahim Ali, Nobumichi Kobayashi

**Affiliations:** 1 Department of Microbiology, Mymensingh Medical College, Mymensingh 2200, Bangladesh; drsangjukta@gmail.com (S.R.); nasreenm19@gmail.com (S.A.N.); nomaan.dr.mmc@gmail.com (M.S.H.); drnaziahaque@gmail.com (N.H.); dr.jkchaity1986@gmail.com (J.K.); drsathi.dmc@gmail.com (F.A.S.); shashwatapaul96@gmail.com (S.P.); 2 Department of Hygiene, Sapporo Medical University School of Medicine, Sapporo 060-8556, Japan; meijisoeaung@sapmed.ac.jp; 3 Netrokona Medical College, Netrokona 2400, Bangladesh; drshyamal10@yahoo.com; 4 Department of Radiology and Imaging, Mymensingh Medical College, Mymensingh 2200, Bangladesh; drnazmul67@gmail.com; 5 Department of Medicine, Mymensingh Medical College, Mymensingh 2200, Bangladesh; drtridipkanti@gmail.com; 6 Department of Urology, Mymensingh Medical College, Mymensingh 2200, Bangladesh; ibrahimurology@gmail.com

**Keywords:** *Enterococcus faecalis*, *Enterococcus faecium*, antimicrobial resistance, VRE, *vanA*, *optrA*, ST

## Abstract

Background/Objectives: *Enterococcus* is one of the major nosocomial pathogens. The present status of antimicrobial resistance determinants and virulence factors was analyzed for current *Enterococcus* causing infectious diseases in Bangladesh. Methods: Clinical isolates of *Enterococcus* recovered from various specimens in a tertiary care hospital were analyzed. Antimicrobial susceptibility was measured by a broth microdilution test, and resistance genes/virulence factors were detected by uniplex/multiplex PCR, along with sequencing analysis as required. The sequence type (ST) of *E. faecalis* and *E. faecium* was identified based on a multilocus sequence typing (MLST) scheme. Results: For a one-year period, a total of 143 isolates (135 *E. faecalis*, 7 *E. faecium*, and 1 *E. hirae*) were collected. Although all *E. faecalis* isolates were susceptible to penicillin, high resistance rates were noted against erythromycin (87%) and levofloxacin (62%). High-level resistance to gentamicin was detected in 30% of *E. faecalis* and 86% of *E. faecium*. Vancomycin resistance due to *vanA* was identified in one isolate each of *E. faecalis* (ST1912, CC116) and *E. faecium* (ST80, CC17). Three *E. faecalis* isolates (2.2%) with ST32 or ST1902 were resistant to linezolid, harboring *optrA-fexA*. Conclusions: The present study identifies the vancomycin-resistant *Enterococcus* harboring *vanA* from humans in Bangladesh and shows the potential spread of *optrA* in multiple lineages of *E. faecalis*.

## 1. Introduction

Enterococci constitute the normal flora of the gastrointestinal tract of humans and thus were previously considered less virulent microorganisms with little clinical importance. However, over the past few decades, enterococci have been emerging as a remarkable pathogen that cause opportunistic, healthcare-associated infections, associated with the acquisition of resistance to multiple antimicrobials [1,2]. In addition to endocarditis, which is the oldest described disease, enterococci cause infections in various sites in the human body, including the urinary tract, intra-abdominal organs, soft tissue, the bloodstream, etc. The increase in nosocomial infections due to enterococci is related to the progress of current medical practices worldwide. The growing number of hospitalized patients who are immunosuppressed, mechanically compromised with catheters, and receiving multiple antimicrobials are considered the background of the spread of drug-resistant opportunistic pathogens like *Enterococcus*.

Acquired resistance to glycopeptides, penicillin, and aminoglycosides (high level) have been known as the major traits of nosocomial strains of *E. faecalis*/*E. faecium* [3]. Currently, the occurrence of resistance to newly introduced antimicrobials, such as linezolid, daptomycin, and tigecycline, is also recognized for its clinical importance [3,4,5]. A meta-analysis revealed that the resistance rates of *E. faecalis* to most antimicrobials increased over time globally [6], and another study showed the highest level of resistance to vancomycin and linezolid in *E. faecalis* and *E. faecium*, respectively, in Southeast Asia compared with other WHO regions [7].

The main mechanism of glycopeptide (e.g., vancomycin) resistance is the alteration of the peptidoglycan synthesis pathway mediated by the *van* operon located on a transposon that contains a key ligase gene, which has been differentiated into *vanA*, *vanB*, *vanC*, *vanD*, *vanE*, *vanG*, *vanL*, *vanM*, and *vanN* [8]. Among these ligase genes, *vanA* and *vanB* are the most commonly detected ones in clinical isolates of vancomycin-resistant enterococci (VRE), conferring different levels of susceptibility to vancomycin and teicoplanin, with transferable traits. High-level resistance to vancomycin is conferred by *vanA*, *vanB*, *vanD* (*E. faecium*), and *vanM* (*E. faecium*), with variable resistance levels for *vanB* and *vanD* depending on the strain. Other *van* gene operons mediate a low to intermediate level of vancomycin resistance.

Linezolid is an antibiotic belonging to the class of oxazolidinone and is used for treatment of infections due to VRE as well as methicillin-susceptible/resistant *S. aureus* and *S. pneumoniae* [9]. Resistance to linezolid has been revealed to be attributable to different mechanisms: mutations in 23S rRNA and *rplC*/*rplD*, the acquisition of the *optrA* or *poxtA* encoding ATP-binding cassette (ABC)-F protein, and a *cfr* variant encoding 23S rRNA methyltransferase [5]. Among them, *optrA*, which is located in plasmids or chromosomes, is the most prevalent [10,11], and its spread has been a concern for public health.

As determinants that facilitate the infection of *Enterococci* to hosts, various virulence factors have been described, including aggregation substance, gelatinase, cytolysin, etc. [12,13]. Virulence traits due to the virulence factors include adherence and invasion to host tissues, abscess formation, the modulation of host inflammatory response, and so forth. The prevalence of the virulence factors is variable depending on strains, and the virulence traits are transmissible among strains [12,13,14]. Accordingly, to monitor the prevalence of virulence factors is of value to predict the clinical impact of *Enterococcus* circulating in healthcare settings.

In Asia, South Asia (India and Pakistan) is shown as the second most prevalent area of VRE, following the western Asian region [15]. In India, the overall prevalence of VRE from human samples has been reported as 12.4%, associated with its increase from the period 2000–2010 (4.8%) to 2011–2020 (14%) [16]. Particularly in the south Indian state, the higher prevalence of vancomycin resistance (43% in *E. faecalis* and 35% in *E. faecium*) due mostly to *vanA* has been described [17]. In Bangladesh, enterococci have been documented as one of the major pathogens of urinary tract infections [18,19] and were totally susceptible to vancomycin until recently [20,21], while the information for human clinical isolates is not sufficient. In contrast, VRE in Bangladesh has been reported among isolates from healthy chicken and raw seafood at low rates [22,23] and also retail meat and rhesus macaques with considerable prevalence [24,25]. Despite the lower prevalence than vancomycin resistance, linezolid-resistant enterococci is found in India [26,27] and was also found in our study in Bangladesh [21]. We previously analyzed clinical isolates of *E. faecalis* in the north-central region of Bangladesh for their antimicrobial resistance and determinants, detecting *optrA*-positive isolates [21]. The present study was conducted as a successive investigation on clinical isolates of enterococci, along with virulence factors, to describe the trend of molecular epidemiological features of antimicrobial-resistant strains.

## 2. Results

### 2.1. Enterococcus Isolates and Antimicrobial Susceptibility

During the one-year study period, a total of 143 enterococcal isolates were recovered from clinical specimens of patients. Among the specimens, urine was the most common (*n* = 127), followed by sputum (*n* = 8), pus (surgical operation site) (*n* = 5), and blood (*n* = 3). Patients were aged 0–88 years (median; 44), with almost same frequency being male (*n* = 73) and female (*n* = 74). Inpatients accounted for 78% (*n* = 111). Enterococcal species identified were *E. faecalis* (*n* = 135), *E. faecium* (*n* = 7), and *E. hirae* (*n* = 1).

Resistance rates to antimicrobials are summarized in Table 1. Both *E. faecalis* and *E. faecium* showed high resistance rates (>61%) to erythromycin and levofloxacin. *E. faecium* showed significantly higher resistance rates to gentamicin (high level) and rifampicin than *E. faecalis* (*p* < 0.01). Against trimethoprim–sulfamethoxazole, resistance levels of both species were similar. All the *E. faecalis* isolates showed susceptibility to penicillin and ampicillin, unlike *E. faecium* isolates that were totally resistant. Resistance to vancomycin and teicoplanin was detected in one isolate each of *E. faecalis* and *E. faecium* (overall rate: 1.4%). Three *E. faecalis* isolates showed resistance to linezolid with an MIC of 8 μg/mL. These isolates were susceptible to tedizolid, while showing resistance to florfenicol and chloramphenicol.

### 2.2. Genotypes and Antimicrobial Resistance Genes of E. faecalis

Sequence type (ST) based on a multilocus sequence typing (MLST) scheme was determined for a total of 35 representative *E. faecalis* isolates, which were selected among those derived from different specimens showing different patterns of antimicrobial resistance. For these isolates, the presence of antimicrobial resistance genes was analyzed by PCR and also sequencing when necessary (Table 2). Among the 14 STs identified, 4 STs (ST1902, ST1912, ST1913, and ST1914) were novel types assigned for 11 isolates in this study. ST1912, belonging to CC116, was detected in eight isolates.

In this study, only the *vanA* gene was detected in VRE, while *vanB*, *vanD*, and *vanG* were negative. Vancomycin-resistant *E. faecalis* (ME-140) was isolated from blood of a neonate, belonged to ST1912, and harbored *vanA* and other resistance genes and six virulence factors, showing resistance to multiple antimicrobials.

Three linezolid-resistant isolates were derived from urine and pus (gallbladder operation site) and belonged to ST32 (two isolates, ME-96 and ME-121) and ST1902 (ME-134), respectively. These isolates had *optrA* located adjacent to the fenicol resistance gene *fexA*, which was revealed by sequencing analysis. Nucleotide sequences of the *optrA-fexA* cluster of ME-96 and ME-121 were identical and showed 100% identity to those of *E. faecalis* strains SF35 (ST330, chicken) and E419 (ST480, human) reported in China [28]. The *optrA-fexA* cluster sequence of ME-134 had 99.89% identity to those of ME-96 and ME-121, and also high identity (>99.8%) to those in plasmids of *E. faecalis* strains M6-97, 452115, and E121 reported previously (Table S1) [10,28,29]. However, this genetic cluster of ME-96, ME121, and ME134 showed 99.1% identity to that of strain SJ82 (ST902), which was previously isolated in the same study site in Bangladesh [21]. The slight difference in the *optrA-fexA* cluster was located in the sequence between *optrA* and *fexA* (approx. 680 bp) (96.65%), while *optrA* and *fexA* were almost identical (>99.5%) (Tables S2–S4). By comparison with the OptrA wild type, its amino acid sequences of ME-96 and ME-121 were assigned to the KD variant (T112K, Y176D), while ME-134 was assigned to the RDK variant (T104R, Y176D, E256K), according to the classification described previously [30].

These linezolid-resistant *E. faecalis* harbored a smaller number of resistance genes (*erm(B)* and *aph(3′)-IIIa*) and virulence factors (*efaA* and *gelE*). Resistance to five or more different classes of antimicrobials was found in ST28, ST179, ST946, and ST1912, most of which had four or more virulence factors. Though ST919 isolates showed susceptibility to most of the antimicrobials, except for erythromycin, they harbored 4–6 virulence factors, including *esp*. Three or four isolates belonged to ST28, ST76, and ST946, which showed different profiles of antimicrobial resistance genes and virulence factors.

### 2.3. Genotypes and Antimicrobial Resistance Genes of E. faecium

Seven isolates of *E. faecium* were classified into four STs, all of which belonged to CC17 (Table 3). An ST80 isolate (ME-141) from sputum harbored *vanA* and showed resistance to multiple antimicrobials, including vancomycin and teicoplanin. Nucleotide sequence of *vanA* of ME-141 was identical to that of *E. faecalis* isolate ME-140 and also to those of VRE registered in GenBank database (e.g., *E. faecalis* strain R712 and *E. faecium* strain V24, accession nos. CP036247 and KX574671, respectively). The macrolide resistance gene *msrC* was detected in all the isolates, while *erm(B)* was found in only two ST80 isolates, including the VRE. Among some aminoglycoside modifying enzyme genes identified, *aac(6′)-Ie-aph(2″)-Ia* was most commonly detected. Although *efaA* was positive in all the *E. faecium*, only ST80 isolates harbored *esp*, and other isolates had *hyl*.

## 3. Discussion

The present study revealed the prevalence of antimicrobial resistance and genetic characteristics of current enterococcus clinical isolates in Bangladesh. A notable finding was the identification of VRE with *vanA* for the first time from humans in this country, though previous studies reported the total susceptibility of *Enterococcus* clinical isolates to vancomycin [20,21,31]. In our study, an overall detection rate of VRE was 1.4% (2/143) among enterococci, with those in *E. faecalis* and *E. faecium* being 0.7% (1/135) and 14% (1/7), respectively. The relative isolation rates in the two species were in line with the global prevalence of VRE from blood, showing a much higher rate in *E. faecium* (10%) than in *E. faecalis* (1.6%) [7]. Originally, South Asia (Southeast Asia by the WHO) has been known to be the region having higher levels of VRE prevalence [7], showing a detection rate of 6.5–16% with an increasing trend in more recent years in India [15,16,32,33,34,35]. In Bangladesh, before the detection of VRE in clinical isolates in this study, VRE was isolated from animal and retail meat [22,24,25], suggesting the spread of VRE in the environment of people’s daily life. This may provide a chance for the transmission of VRE to humans, along with selection with vancomycin in medical care facilities. In our present study, 78% of enterococcal isolates were derived from inpatients, including those with resistance to vancomycin or linezolid. Thus, the etiologic enterococci are more likely to be transmitted in the hospital. One of the VRE isolates (*E. faecalis*) was recovered from the blood of a neonate in a neonatology department where vancomycin is administered as required. This may indicate the need for more attention to VRE due to hospital-acquired infection in wards where vancomycin is used, although the prevalence of VRE in Bangladesh still seems to be low.

In accordance with the dominance of *vanA* in VRE in epidemiological studies in India [17,33,34,36], only *vanA* was identified in our study, in two VRE, ST80 (CC17) *E. faecium* and ST1912 (CC116) *E. faecalis*. ST80-*vanA E. faecium* was recently described as the dominant VRE in Denmark [37], Thailand [38], and southern China [39], while ST80 is rarely found in the other provinces in China, with the most common type being ST78 [40,41,42]. Though it is not certain whether ST80 is increasing regionally, the detection of ST80-*vanA E. faecium* in Bangladesh may suggest its original prevalence in a wide global area or spread after its emergence. Because CC17 is known as the major nosocomial clone of *E. faecium* worldwide [43], ST80 is considered possible to occur anywhere as a further hospital-adapted variant. 

Among the *E. faecalis* clones analyzed in the present study, a novel type, ST1912, was detected in eight isolates, among which one isolate was VRE. This ST belongs to CC116, which includes some STs (ST946 and ST947) detected in the same study site in 2018 [21]. CC116 is one of the major clonal groups of *E. faecalis* [44], with ST116 being detected in Europe, Africa, Asia, and South America. Thus, CC116 is considered a globally distributed lineage. In our study site, this lineage seems to be prevalent persistently, and a part of the strain might have acquired *vanA*, as observed in this study.

Although the prevalence of linezolid resistance is reported to be less than vancomycin resistance (overall rates of 0.9% for both *E. faecalis* and *E. faecium* blood isolates), linezolid resistance has been increasing globally and regionally [6,45], and *optrA* is the most responsible determinant among *E. faecalis* [7]. The Southeast Asia region was shown to have a slightly higher rate of linezolid resistance [7], and in Asia, most of the resistant isolates were reported in China [26]. In a study in India, linezolid resistance was found in 2.8% of enterococcus clinical isolates (*E. faecium*) in a tertiary care hospital, and most isolates harbored *optrA* [27]. In our previous study in Bangladesh, 2.4% (five isolates) of *E. faecalis* from urinary tract infections showed non-susceptibility to linezolid (MIC, 4 μg/mL), having *optrA* and belonging to CC59 (ST59 and ST917) and ST902 [21]. Although linezolid resistance was detected in the present study in three isolates of *E. faecalis* (2.2%) with *optrA*, their genotypes (ST32 and ST1902) were distinct from the previous study. To date, linezolid-resistant *E. faecalis* with *optrA* have been identified in various STs, including more common types such as ST16, ST116, ST256, and ST480 [10,28,46,47,48]. In the PubMLST database, ST32 (*n* = 16) was mostly derived from animals (poultry and pigs) in Europe, Africa, and Asia and was described as an *optrA*-positive isolate from pigs [49]. ST1902 is an SLV of ST941, involving five related STs that may form CC941, while STs of this group have not been described as a linezolid-resistant strain to date. The identification of two new lineages, in addition to previously detected ones, may suggest the potential spread of *optrA*-positive *E. faecalis*.

ST32 and ST1902 *E. faecalis* isolates had different genetic features, i.e., a slight sequence difference in the *optrA-fexA* cluster and having a distinct OptrA variant, i.e., the KD and RDK variant, respectively. These findings indicated that ST32 and ST1902 have *optrA* from different origins. The KD and RDK variants were shown to be common in isolates from patients and healthy individuals in China, respectively [30,47], and both are associated with a higher MIC of linezolid. In contrast, *optrA*-positive *E. faecalis* previously isolated in Bangladesh had an EDD variant with a lower linezolid MIC value [21], implying that more resistant clones have been introduced more recently to clinical settings. Although the origin of ST32 is not evident, its obvious prevalence in animals [49] suggests that it might be derived from animals (e.g., chicken and pig) via direct contact or retail meat. Actually, the presence of linezolid-resistant enterococci was detected in animals in Bangladesh [24,25]. The occurrence of *optrA*-positive, linezolid-resistant enterococcus has been noted in patients without exposure to linezolid [26]. There is a similar situation in Bangladesh, in our study setting, because the use of linezolid is still limited in hospitals for advanced treatment. A possible reason for the spread of the *optrA*-positive *E. faecalis* may be selection by co-resistance to other antimicrobials, such as macrolides. In the present study, *optrA*-positive isolates were resistant to erythromycin, having *erm(B)*. Therefore, the use of erythromycin for treatment of other infections could select *optrA*-positive clones. The possibility of such co-selection of multiple drug resistance may be a key challenge in the control of antimicrobial resistance in bacteria that commonly inhabit humans and livestock.

Pathogenic enterococci in humans are known to produce several specific virulence factors [14,50], though their prevalence is different depending on study settings/patients. As reported in other studies, *E. faecalis* clinical isolates harbor *ace*, *efaA*, *asa1*, and *gelE* at high rates [13,42,50,51], while the prevalence of *esp* in *E. faecalis* is lower and variable (44–63%). Esp is a large cell surface protein encoded by a pathogenicity island and has a pathogenic role by strengthening biofilm in host environments [52], with higher prevalence in *E. faecium* than *E. faecalis* [42,51]. Notably, a high detection rate of *esp* was reported for VRE of both species [12,40,53], as observed for the VRE isolated in our study. Because the prevalence of *esp* may be of significance to generally understand the virulence status of enterococci, this gene should be further monitored for clinical isolates, and the biofilm formation capacity of the bacterial cells should be studied.

The hyaluronidase gene (*hyl*) is also specified as a virulence factor of vancomycin-resistant *E. faecium* with a lower prevalence than *esp* [12,40,51,53], while being positive in vancomycin-susceptible isolates in the present study. In contrast, the *optrA*-positive *E. faecalis* had fewer virulence factors (only *efaA* and *gelE*), lacking *esp* in our study, suggesting its lower pathogenicity. Although little is known about the virulence factors in linezolid-resistant enterococci, the virulence of *optrA*-positive enterococci is presumed to be generally low, because such strains were detected in healthy human populations [30], and their major isolation sources were urine and stool (>60%) [26,27,46]. However, *optrA*-positive enterococci have been also isolated from blood (17–21% [26]) and sites of invasive infections [47], suggesting its increased virulence in a part of the strains. Further study is necessary to clarify the prevalence of virulence factors in linezolid-resistant enterococci from infections in humans and its changing trend. As a limitation of the present study, the number of *E. faecium* isolates was too low to evaluate the trend of antimicrobial resistance in the country appropriately. Further large-scale surveillance is required to determine this point.

## 4. Materials and Methods

### 4.1. Bacterial Isolates

As the isolation source of enterococci, all types of specimens were collected from inpatients and outpatients in the Mymensingh Medical College (MMC) hospital, Mymensingh, Bangladesh, consecutively, for a one-year period from February 2023 to January 2024. From one patient, only one isolate was included in this study. Bacterial culture was performed using a chromogenic agar plate (HiCrome UTI Agar, HiMedia Laboratories, Thane, India), followed by incubation aerobically at 37 °C for 48 h. *Enterococcus*-like colonies on the agar plates were picked up and further examined by Gram staining and biochemical tests. To identify *E. faecalis* and *E. faecalis*, PCR targeting the PBP5 gene was performed for all the isolates [54], and species of some isolates were identified by the determination of the 16S rRNA gene sequence, as previously described [55]. The obtained isolates were stored in Microbank (Pro-Lab Diagnostics, Richmond Hill, ON, Canada) at −80 °C and were recovered when they were analyzed.

### 4.2. Antimicrobial Susceptibility Testing

Antimicrobial susceptibility was measured by the broth microdilution test using Dry Plate Eiken DP42 (Eiken, Tokyo, Japan). This microtiter plate includes the following antimicrobials with a range of concentrations (mg/L) for testing: penicillin (PEN, 0.12–8), ampicillin (AMP, 0.25–8), ampicillin–sulbactam (SAM, 1/2–8/16), imipenem (IPM, 0.25–8), high-level gentamicin resistance (GEN-HLR, 500), minocycline (MIN, 1–8), erythromycin (ERY, 0.25–4), levofloxacin (LVX, 0.25–4), linezolid (LZD, 0.5–4), rifampicin (RIF, 0.5–2), daptomycin (DAP, 0.25–4), teicoplanin (TEC, 0.5–16), and vancomycin (VAN, 0.5–16). For the isolates with LZD resistance, the MICs of chloramphenicol (CHL), florfenicol (FFC), and tedizolid (TDZ) were determined by the broth microdilution method. Susceptibility/resistance was judged according to the break points mentioned in the CLSI and EUCAST guidelines [56,57]. For CHL and FFC, susceptibility was interpreted by MIC as described previously [58].

### 4.3. Detection of Antimicrobial Resistance Genes and Virulence Factors

According to the previously described primers and conditions [59], uniplex or multiplex PCR assays were used to detect the following antimicrobial resistance genes: beta-lactamase gene, *blaZ*; aminoglycoside modifying enzymes (AME) genes, *aac(6′)-Ie-aph(2″)-Ia*, *aph(3′)-IIIa*, *ant(6)-Ia*, *ant(4′)-Ia*, *aph(2″)-Id/Ie*, and *ant(9)-Ia*; macrolide resistance genes, *erm(A)*, *erm(B)*, *erm(C)*, *erm(T)*, *msr(A)*, *msr(B),* and *msr(C)*; vancomycin resistance genes, *vanA*, *vanB*, *vanD*, *vanE*, and *vanG*; tetracycline resistance genes, *tet(L)*, *tet(M)*, *tet(K)*, *tet(O)*, *tet(S)*, *tet(T)*, and *tet(U)*; oxazolidinone and fenicol resistance genes, *optrA*, *poxtA*, and *cfr*; and the phenicol exporter gene, *fexA*. Similarly, the previously reported PCR methods [13,14] were employed to detect the virulence factors *asa1* (aggregation substance), *cylA* (cytolysin activator), *efaA* (endocarditis antigen/cell wall adhesin), *gelE* (gelatinase), *esp* (surface protein), *ace* (collagen-binding protein), and *hyl* (hyaluronidase).

### 4.4. Sequence Analysis of vanA and optrA-fexA Clusters

Nucleotide sequences of the *vanA* and *optrA-fexA* clusters were determined by PCR and direct sequencing using the BigDye Terminator v. 3.1 Cycle Sequencing Kit (Applied Biosystems, Foster City, CA, USA) on an automated DNA sequencer (ABI PRISM 3100, Thermo Fisher Scientific, Tokyo, Japan). The primers used for sequencing are shown in Table S5. The multiple alignment of the nucleotide and deduced amino acid sequences and calculation of identity were performed by using the Clustal Omega program (https://www.ebi.ac.uk/jdispatcher/msa/clustalo?outfmt=fa) (accessed on 10 January 2025).

### 4.5. Multilocus Sequence Typing (MLST)

For selected isolates of *E. faecalis* having different drug resistance profiles, and all the isolates of *E. faecium*, the sequence type (ST) was identified by sequencing of 7 loci based on the MLST schemes [60,61] using the web-based genotyping tool PubMLST (https://pubmlst.org/efaecalis/) (accessed on 15 December 2024). The clonal complex (CC) of ST was further assigned by a BURST analysis available on the PubMLST website.

### 4.6. GenBank Accession Numbers

The nucleotide sequences of the *vanA* and *fexA–optrA* clusters were deposited in the GenBank database under the accession numbers listed in Table S6.

### 4.7. Statistical Analysis

The difference in the prevalence of antimicrobial resistance between *E. faecalis* and *E. faecium* was statistically analyzed by Fisher’s exact test using the js-STAR XR ver.1.1.9 software (https://www.kisnet.or.jp/nappa/software/star/index.htm, accessed on 20 December 2024). A *p*-value < 0.01 was considered statistically significant.

## Figures and Tables

**Table 1:** Antimicrobial resistance rates among *E. faecalis*, *E. faecium*, and *E. hirae* isolates identified in this study.

Antimicrobials	*E. faecalis n* = 135 (%)	*E. faecium n* = 7 (%)	*E. hirae n* = 1 (%)
Penicillin	0 (0)	7 (100) *^1^	0 (0)
Ampicillin	0 (0)	7 (100) *^1^	0 (0)
Ampicillin-sulbactam	0 (0)	7 (100) *^1^	0 (0)
Imipenem	0 (0)	7 (100) *^1^	0 (0)
Minocycline	17 (12.6)	3 (42.9)	0 (0)
Erythromycin	118 (87.4)	7 (100)	0 (0)
Levofloxacin	83 (61.5)	7 (100)	0 (0)
Rifampicin	22 (16.3)	7 (100) *^1^	0 (0)
High-level resistance to Gentamicin	40 (29.6)	6 (85.7) *^1^	0 (0)
Arbekacin	114 (84.4)	7 (100)	0 (0)
Trimethoprim-sulfamethoxazole	45 (28.1)	2 (28.6)	0 (0)
Fosfomycin	2 (1.5)	0 (0)	0 (0)
Linezolid	3 (2.2)	0 (0)	0 (0)
Vancomycin	1 (0.7)	1 (14.3)	0 (0)
Teicoplanin	1 (0.7)	1 (14.3)	0 (0)

*^1^ *p* < 0.01.

**Table 2:** **ST, virulence factors, resistance profiles and antimicrobial resistance genes in the selected *E. faecalis* isolates.**

Isolate ID	Age	Sex	Specimen	Ward *^1^	ST *^2^ (CC)	Allelic Profile	Virulence Gene Profile	Resistance Profile *^3^	Drug Resistance Determinants
ME-77	23	F	Urine	OPD	ST16	5-1-1-3-7-7-6	*asa1, cylA, efaA, esp, ace*	ERY, RIF, ABK	*erm(B)*, *aac(6′)-Ie-aph(2″)-Ia*
ME-80	20	F	Urine	Gynaecology	ST23	2-3-13-11-3-2-2	*asa1, cylA, efaA, ace*	RIF, ABK	
ME-24	28	F	Urine	Gynaecology	ST28	4-4-8-3-8-1-3	*asa1, gelE, esp, ace*	LVX, RIF, ABK	
ME-138	65	F	Sputum	Medicine	ST28	4-4-8-3-8-1-3	*asa1, gelE, esp, ace*	MIN, ERY, LVX, HLG, ABK, SXT	*erm(B)*, *aac(6′)-Ie-aph(2″)-Ia*, *aph(3′)-IIIa*
ME-116	65	M	Urine	Urology	ST28	4-4-8-3-8-1-3	*asa1, cylA, efaA, gelE, ace*	MIN, ERY, LVX, ABK, SXT	*erm(B)*, *aph(3′)-IIIa*
ME-146	55	F	Sputum	Medicine	ST28	4-4-8-3-8-1-3	(-)	ERY, LVX, SXT	
ME-96	40	F	Urine	Urology	ST32	8-7-9-5-4-4-1	*efaA, gelE*	ERY, LZD, ABK, FFC, CHL	*optrA, fexA, erm(B), aph(3′)-IIIa, ant(6)-Ia*
ME-121	52	M	Urine	Urology	ST32	8-7-9-5-4-4-1	*efaA, gelE*	ERY, LZD, ABK, LVX, FFC, CHL	*optrA, fexA, erm(B), aph(3′)-IIIa, ant(6)-Ia*
ME-110	75	F	Urine	Urology	ST76	22-6-7-26-22-4-4	*asa1, cylA, efaA*	ERY, LVX, ABK	
ME-144	16	F	Sputum	Medicine	ST76	22-6-7-26-22-4-4	*gelE, esp, ace*	MIN, ERY, LVX, SXT	
ME-11	8	M	Urine	Urology	ST76	22-6-7-26-22-4-4	*efaA, esp, ace*	ABK	
ME-65	2	M	Urine	OPD	ST116	17-2-22-1-14-14-1	*efaA, gelE, ace*	MIN, ERY, LVX, RIF, ABK	*erm(B)*
ME-63	6	M	Urine	Medicine	ST116	17-2-22-1-14-14-1	*gelE, ace*	ERY, LVX, RIF, ABK	*erm(B)*
ME-34	26	F	Urine	Gynaecology	ST179	5-1-1-3-7-1-6	*esp, ace*	ERY, LVX, RIF, HLG, ABK, SXT	*erm(B)*, *aac(6′)-Ie-aph(2″)-Ia*, *aph(3′)-IIIa*
ME-139	40	F	Wound	Surgery	ST919	17-4-8-3-14-1-1	*asa1, cylA, efaA, gelE, esp, ace*	ERY, LVX, ABK	
ME-93	55	F	Urine	Urology	ST919	17-4-8-3-14-1-1	*asa1, cylA, efaA, gelE, esp, ace*	ERY	
ME-95	70	M	Urine	Urology	ST919	17-4-8-3-14-1-1	*asa1, cylA, efaA, gelE, esp, ace*	ERY	*erm(B)*
ME-149	25	M	Pus	Surgery	ST919	17-4-8-3-14-1-1	*asa1, efaA, gelE, esp, ace*	ERY, ABK	*erm(B)*
ME-58	4	F	Urine	OPD	ST919	17-4-8-3-14-1-1	(-)	ERY	
ME-133	60	M	Sputum	Medicine	ST946	12-4-22-1-14-14-1	*asa1, cylA, efaA, gelE, esp*	ERY, LVX, RIF, HLG, ABK, SXT	*erm(B)*, *aac(6′)-Ie-aph(2″)-Ia*, *aph(3′)-IIIa, ant(9)-Ia*
ME-42	35	F	Urine	Gynaecology	ST946	12-4-22-1-14-14-1	*gelE, esp*	MIN, ERY, LVX, HLG, ABK, SXT	*erm(B)*, *aac(6′)-Ie-aph(2″)-Ia*, *aph(3′)-IIIa*
ME-82	50	F	Urine	Gynaecology	ST946	12-4-22-1-14-14-1	*asa1*	ERY, LVX, HLG, ABK, SXT	*aac(6′)-Ie-aph(2″)-Ia*, *aph(3′)-IIIa*
ME-102	39	M	Urine	Urology	ST946	12-4-22-1-14-14-1	*asa1, cylA, efaA, gelE, esp, ace*	ERY, LVX, HLG, ABK	*erm(B)*, *aac(6′)-Ie-aph(2″)-Ia*, *aph(3′)-IIIa*
ME-22	8	M	Urine	OPD	ST1416	12-7-7-17-2-2-5	*efaA, gelE*	ABK	
ME-134	60	M	Pus (operation site)	Surgery	**ST1902**	9-2-7-16-11-11-8	*efaA, gelE, ace*	ERY, LZD, ABK, FFC, CHL	*optrA, fexA, erm(B), aph(3′)-IIIa, ant(9)-Ia*
ME-140	25 days	M	Blood	Neonatology	**ST1912** (CC116)	4-4-22-1-14-1-1	*asa1, cylA, efaA, gelE, esp, ace*	PEN, AMP, ERY, LVX, HLG, ABK, SXT, FOF, VAN, TEC	*vanA*, *erm(B)*, *aac(6′)-Ie-aph(2″)-Ia*, *aph(3′)-IIIa*
ME-1	40	M	Urine	Medicine	**ST1912** (CC116)	4-4-22-1-14-1-1	*asa1, cylA, efaA, gelE, esp, ace*	MIN, ERY, LVX, HLG, ABK, SXT	*erm(B)*, *aac(6′)-Ie-aph(2″)-Ia*, *aph(3′)-IIIa*
ME-9	35	F	Urine	Urology	**ST1912** (CC116)	4-4-22-1-14-1-1	*asa1, cylA, efaA, gelE, esp, ace*	ERY, LVX, HLG, ABK, SXT	*erm(B)*, *aac(6′)-Ie-aph(2″)-Ia*, *aph(3′)-IIIa*
ME-143	85	M	Sputum	Medicine	**ST1912** (CC116)	4-4-22-1-14-1-1	*asa1, efaA, gelE, esp, ace*	MIN, ERY, LVX, RIF, HLG, ABK, SXT	*erm(B)*, *aac(6′)-Ie-aph(2″)-Ia*, *aph(3′)-IIIa*
ME-145	1	F	Blood	Paediatrics	**ST1912** (CC116)	4-4-22-1-14-1-1	*asa1, efaA, gelE, esp, ace*	MIN, ERY, LVX, RIF, HLG, ABK, FOF	*erm(B)*, *aac(6′)-Ie-aph(2″)-Ia*, *aph(3′)-IIIa*
ME-15	4	M	Urine	Urology	**ST1912** (CC116)	4-4-22-1-14-2-1	*asa1, cylA, gelE, esp, ace*	ERY, LVX, RIF, ABK	*erm(B)*
ME-14	7	M	Urine	Urology	**ST1912** (CC116)	4-4-22-1-14-1-1	*asa1, cylA, efaA, gelE, esp*	MIN, ERY, LVX, HLG, ABK, SXT	*erm(B)*, *aac(6′)-Ie-aph(2″)-Ia*, *aph(3′)-IIIa*
ME-125	24	M	Urine	Medicine	**ST1912** (CC116)	4-4-22-1-14-1-1	*asa1, cylA, efaA, gelE*	ERY, ABK	
ME-83	18	F	Urine	Medicine	**ST1913**	12-4-22-37-14-2-1	*asa1*	ERY, RIF, ABK	*erm(B)*, *aph(3′)-IIIa*
ME-56	3	M	Urine	OPD	**ST1914** (ST919 SLV)	17-4-4-3-14-1-1	*gelE, esp, ace*	ERY	

*^1^ OPD, outpatient department; *^2^ Boldface shows newly identified ST in this study. CC, clonal complex; SLV, single-locus variant. *^3^ Abbreviations: ABK, arbekacin; AMP, ampicillin; CHL, chloramphenicol; ERY, erythromycin; FFL, florphenicol; FOF, osfomycin; HLG, gentamicin (high-level); LVX, levofloxacin; LZD, linezolid; PEN, penicillin; RIF, rifampicin; SXT, trimethoprim/sulfamethoxazole; TEC, teicoplanin; VAN, vancomycin.

**Table 3:** ST, resistance profiles, antimicrobial resistance genes, and virulence factors in *E. faecium* isolates.

Isolate ID	Age	Sex	Sample type	Ward	ST (CC)	Allelic profile	Virulence Gene Profile	Resistance Profile	Drug Resistance Determinants
ME-141	56	M	Sputum	Medicine	ST80 (CC17)	9-1-1-1-12-1-1	*esp*, *efaA*	PEN, AMP, SAM, IMP, RIF, MIN, ERY, LVX, HLG, ABK, SXT, FOF, VAN, TEC	*vanA*, *erm(B)*, *msrC*, *aac(6′)-Ie-aph(2″)-Ia*, *aph(3′)-IIIa*
ME-13	23	F	Urine	Urology	ST80 (CC17)	9-1-1-1-12-1-1	*esp*, *efaA*	PEN, AMP, SAM, IMP, RIF, ERY, LVX, HLG, ABK	*msrC, aph(3′)-IIIa*
ME-91	88	M	Urine	Medicine	ST80 (CC17)	9-1-1-1-12-1-1	*esp*, *efaA*	PEN, AMP, SAM, IMP, RIF, ERY, LVX, HLG, ABK	*erm(B)*, *msrC*, *aac(6′)-Ie-aph(2″)-Ia*, *aph(3′)-IIIa, ant(6)-Ia*
ME-137	12 days	F	Blood	Neonatology	ST321 (CC17)	7-3-1-1-1-1-3	*hyl, efaA*	PEN, AMP, SAM, IMP, RIF, ERY, LVX, HLG, ABK	*msrC, aac(6′)-Ie-aph(2″)-Ia, aph(2″)-Id/Ie*
ME-90	32	M	Urine	Medicine	ST1887 (CC17)	5-1-1-1-12-7-1	*hyl, efaA*	PEN, AMP, SAM, IMP, MIN, RIF, ERY, LVX, HLG, ABK	*msrC, aac(6′)-Ie-aph(2″)-Ia*
ME-148	37	M	Pus	Surgery	ST1887 (CC17)	5-1-1-1-12-7-1	*hyl, efaA*	PEN, AMP, SAM, IMP, RIF, ERY, LVX, HLG, ABK	*msrC, aac(6′)-Ie-aph(2″)-Ia*
ME-132	80	M	Urine	Medicine	ST2675 * (CC17)	9-1-1-18-5-1-1	*hyl, efaA*	PEN, AMP, SAM, IMP, RIF, ERY, LVX, ABK	*msrC*

* Novel ST identified in this study.

## Data Availability

Data is contained within the article or Supplementary Material.

## Supplementary Materials

The following supporting information can be downloaded at https://www.mdpi.com/article/10.3390/antibiotics14030261/s1, Table S1: Nucleotide sequence identity of *optrA-fexA cluster*; Table S2: Nucleotide sequence identity of *fexA*; Table S3: Nucleotide sequence identity of sequence between *optrA and fexA*; Table S4: Nucleotide sequence identity of *optrA*; Table S5: Primers used for analysis of *fexA-optrA* and *vanA* identified in this study; Table S6: GenBank accession numbers of *vanA* gene and *optrA-fexA* cluster sequences.
