# Supplementary material for: Isolation of vanA-Mediated Vancomycin-Resistant Enterococcus faecalis (ST1912/CC116) and Enterococcus faecium (ST80/CC17), optrA-Positive Linezolid-Resistant E. faecalis (ST32, ST1902) from Human Clinical Specimens in Bangladesh
Source: Antibiotics (Basel). 2025 Mar 4;14(3):261. doi: 10.3390/antibiotics14030261 (PMC11939402; doi:10.3390/antibiotics14030261)
Supplement: Supplementary file 1 [file antibiotics-14-00261-s001.zip › Table S1-4.pdf]

Table S1 Nucleotide sequence identity of *optrA-fexA* cluster

	Strain							
Strain	SJ82	ME-96	ME-121	ME-134	pWHXH	M6-97	E121	452115
1: SJ82	100.00	99.19	99.19	99.11	99.11	99.11	99.14	99.14
2: ME-96	99.19	100.00	100.00	99.89	99.89	99.89	99.91	99.91
3: ME-121	99.19	100.00	100.00	99.89	99.89	99.89	99.91	99.91
4: ME-134	99.11	99.89	99.89	100.00	100.00	100.00	99.93	99.93
5: pWHXH	99.11	99.89	99.89	100.00	100.00	100.00	99.93	99.93
6: M6-97	99.11	99.89	99.89	100.00	100.00	100.00	99.93	99.93
7: E121	99.14	99.91	99.91	99.93	99.93	99.93	100.00	99.96
8: 452115	99.14	99.91	99.91	99.93	99.93	99.93	99.96	100.00

GenBank accession number of *E. faecalis* strain : CP103863, SJ82 chromosome; PQ118972, ME-96; PQ118973, ME-121; PQ118974, ME-134; MH225422, WHXH plasmid pWHXH; M6-97; KT862776, E121 plasmid pE121; MF443368, 452115 plasmid

Table S2 Nucleotide sequence identity of *fexA*

	Strain							
Strain	pWHXH	ME-96	ME-121	ME-134	E121	M6-97	452115	SJ82
1: pWHXH	100.00	100.00	100.00	100.00	100.00	100.00	100.00	99.51
2: ME-96	100.00	100.00	100.00	100.00	100.00	100.00	100.00	99.51
3: ME-121	100.00	100.00	100.00	100.00	100.00	100.00	100.00	99.51
4: ME-134	100.00	100.00	100.00	100.00	100.00	100.00	100.00	99.51
5: E121	100.00	100.00	100.00	100.00	100.00	100.00	100.00	99.51
6: M6-97	100.00	100.00	100.00	100.00	100.00	100.00	100.00	99.51
7: 452115	100.00	100.00	100.00	100.00	100.00	100.00	100.00	99.51
8: SJ82	99.51	99.51	99.51	99.51	99.51	99.51	99.51	100.00

Table S3 Nucleotide sequence identity of sequence between *optrA* and *fexA*

	Strain							
Strain	pWHXH	ME-96	ME-121	ME-134	E121	M6-97	452115	SJ82
1: pWHXH	100.00	100.00	100.00	100.00	100.00	100.00	100.00	96.65
2: ME-96	100.00	100.00	100.00	100.00	100.00	100.00	100.00	96.65
3: ME-121	100.00	100.00	100.00	100.00	100.00	100.00	100.00	96.65
4: ME-134	100.00	100.00	100.00	100.00	100.00	100.00	100.00	96.65
5: E121	100.00	100.00	100.00	100.00	100.00	100.00	100.00	96.65
6: M6-97	100.00	100.00	100.00	100.00	100.00	100.00	100.00	96.65
7: 452115	100.00	100.00	100.00	100.00	100.00	100.00	100.00	96.65
8: SJ82	96.65	96.65	96.65	96.65	96.65	96.65	96.65	100.00

Table S4 Nucleotide sequence identity of *optrA*

	Strain							
Strain	SJ82	ME-96	ME-121	pWHXH	ME-134	M6-97	E121	452115
1: SJ82	100.00	99.81	99.81	99.66	99.66	99.66	99.71	99.71
2: ME-96	99.81	100.00	100.00	99.76	99.76	99.76	99.81	99.81
3: ME-121	99.81	100.00	100.00	99.76	99.76	99.76	99.81	99.81
4: pWHXH	99.66	99.76	99.76	100.00	100.00	100.00	99.86	99.86
5: ME-134	99.66	99.76	99.76	100.00	100.00	100.00	99.86	99.86
6: M6-97	99.66	99.76	99.76	100.00	100.00	100.00	99.86	99.86
7: E121	99.71	99.81	99.81	99.86	99.86	99.86	100.00	99.90
8: 452115	99.71	99.81	99.81	99.86	99.86	99.86	99.90	100.00
